# From Nanosystems to a Biosensing Prototype for an Efficient Diagnostic: A Special Issue in Honor of Professor Bansi D. Malhotra

**DOI:** 10.3390/bios11100359

**Published:** 2021-09-29

**Authors:** Ajeet Kaushik, Raju Khan, Pratima Solanki, Sonu Gandhi, Hardik Gohel, Yogendra K. Mishra

**Affiliations:** 1 NanoBioTech Laboratory, Department of Environmental Engineering, Florida Polytechnic University, Lakeland, FL 33805, USA; 2 CSIR-Advanced Materials & Processes Research Institute (AMPRI), Bhopal 462026, India; khan.raju@gmail.com; 3 Special Center for Nano Science, Jawaharlal Nehru University, New Delhi 110067, India; pratimarsolanki@gmail.com; 4 DBT-National Institute of Animal Biotechnology (NIAB), Hyderabad 500032, India; gandhi@niab.org.in; 5 Applied Artificial Intelligence Laboratory, University of Houston-Victoria, Victoria, TX 77901, USA; GohelH@uhv.edu; 6 Smart Materials, NanoSYD, Mads Clausen Institute, University of Southern Denmark, Alsion 2, 6400 Sønderborg, Denmark; mishra@mci.sdu.dk

**Keywords:** smart functional materials, green technology, nano-biosensor, miniaturized systems, intelligent health care, personalized health care, POC diagnostics

## Abstract

It has been proven that rapid bioinformatics analysis according to patient health profiles, in addition to biomarker detection at a low level, is emerging as essential to design an analytical diagnostics system to manage health intelligently in a personalized manner. Such objectives need an optimized combination of a nano-enabled sensing prototype, artificial intelligence (AI)-supported predictive analysis, and Internet of Medical Things (IoMT)-based bioinformatics analysis. Such a developed system began with a prototype demonstration of efficient diseases diagnostics performance is the future diseases management approach. To explore these aspects, the Special Issue planned for the nano-and micro-technology section of MDPI’s *Biosensors* journal will honor and acknowledge the contributions of Prof. B.D. Malhotra, Ph.D., FNA, FNASc has made in the field of biosensors.

Recently, due to their success, the Internet of Medical Things (IoMT)-assisted miniaturized biomedical electronics have emerged as a potential analytical tool to manage disease. As a recent example, the infection diagnostics of viral infectious diseases, such as COVID-19, seem manageable due to the collective approach of artificial intelligence (AI) for predictive analysis, IoMT, rapid testing system, performance at Point-of-Care (POC), bioinformatics sharing, along with rapid analytics and timely therapy decisions. An optimized combination of nano-enabled biosensing, POC testing, AI support, and testing interfaced with the IoMT, emerged as very useful, not only for efficient diagnostics but also to make disease management possible at a personalized level (Figure 1). In addition to desired and controlled performance, these systems need to be improved significantly. This can be achieved using the following methods: (1) developing a sensing prototype based on a smart optoelectronic nanosystem to achieve high sensitivity, a low detection limit, and selectivity; (2) using accurate diagnostics without interferents or loss to select a real sample source. These methods validate and scale up the sensor for clinical applications.

Taking the above-discussed technicalities into consideration, and with the aim of developing the intelligent approaches needed for better health, a Special Issue is planned for the nano-and micro-technology section of MDPI’s *Biosensors* journal to honor Prof. B.D. Malhotra, Ph.D., FNA, FNASc in light of his outstanding and highly significant contribution in the field of biosensors. Dr. B.D. Malhotra received his Ph.D. from the University of Delhi in Delhi in 1980. He has published 330 papers in refereed international journals (citations: 23,570; index: 83), has filed 11 patents (in India and overseas), and has co-authored a textbook on Nanomaterials for Biosensors: Fundamentals and Applications and Biosensors: Fundamentals and Applications. He is the recipient of the National Research Development Corporation Award 2005 for the invention of a ‘Blood Glucose Biochemical Analyzer’, was appointed as a Fellow of the Indian National Science Academy by the National Academy of Sciences in India, and is an academician in the Asia Pacific Academy of Materials (APAM). His current research activities include biosensors, POC diagnostics, nano-biomaterials, biofuel cells, ordered molecular assemblies, conducting polymers, Langmuir–Blodgett films, self-assembled monolayers, advanced functionalized nanosystems, hybrid nanosystems, nano-biotechnology, biomedical engineering, and biomolecular electronics.

Since 1994, he has explored functional materials for biosensing applications. He is well known as the father of biosensors in India and as a leading and accomplished scientist at the international level. His efforts initiated biosensing research in India, and as a result, this field is growing rapidly nationwide. His research is multidisciplinary and focuses on detecting targeted biomarkers not only in a physiological range but at a very low level as well. Such systems are emerging as efficient analytical tools to manage disease progression, therapy decisions, and therapy assessments with POC applications. Such approaches are of tunable performance and can be optimized for diagnostics in personalized healthcare settings if supported by AI and the IoMT. Dr. Malhotra is currently a DST-SERB (Govt. of India) Distinguished Fellow and an Adjunct Professor with the Department of Biotechnology at Delhi Technological University in Delhi, India.

*Biosensors* (ISSN 2079-6374, IF:5.519) provides an advanced forum for studies related to the science and technology of biosensors and biosensing. It publishes original research papers, comprehensive reviews, and communications. This journal aims to encourage scientists to publish their experimental and theoretical results in as much detail as possible. In accordance with this mission, the ‘Nano- and Micro-Technologies in Biosensors’ section covers all aspects of research on miniaturized devices in terms of their use in the detection and assay of biological species. The field deals with the design, fabrication, and application of biosensors in the environmental analysis, food, and biopharmaceutical industries, as well as clinical and healthcare technology. The emphasis is on multiplexed and multi-analyte measurement in small samples, especially those involving difficult locations. In this regard, it is expected that many articles describing applications will be centered on the detection of biomarkers and particles, such as bacteria and viruses. Sensor structures are anticipated to include those based on nano- and micro-electrochemical, fiber-optic, lab-on-a-chip, and piezoelectric technologies.

According to the scope of the section and the objectives of this specials issue, this Special Issue will focus on collecting original research and comprehensive review articles based on the following topics:

- Biosensors for metabolite diagnostics;
- Biosensors for agro-food safety and quality assessment;
- Biosensors for cancer diagnostics;
- Biosensors for infectious disease management;
- Lab-on-a-chip supported biosensing systems;
- Microfluidic devices for efficient biosensing;
- Point-of-care biosensing for disease management;
- Efficient biosensing for brain functional assessment;
- Artificial Intelligence (AI) and the Internet of Medical Things (IoMT) for intelligent healthcare;
- Aspects of 3D and green technology for efficient biosensing.

This Special Issue honors the contributions of Prof. B.D. Malhotra has made as a scholar, mentor, supervisor, scientist, and technologist in the field of biosensors for diagnostics applications with the message that systematic, well-planned research is the foundation for developing next-generation analytical, diagnostic tools. Such systems are well-established at the laboratory level, but to make them a part of healthcare management, a sincere focus on interfacing AI and IoMT with the biosensing prototype is a key requirement. With this motivation, this Special Issue invites those experts to explore advanced functionalized nano-enabled biosensing prototypes supported by a miniaturized analyzer for POC application in combination with optimized predictive approaches based on AI and IoMT to achieve intelligent healthcare in a personalized manner [1,2,3,4].

As Prof. B.D. Malhotra says, a lot has been achieved in this direction, and future research has a path to this destination, but well-organized collaborative efforts supported by a public–private partnership are required to achieve these goals.

The recent progress in the field of nano-bio interfaces for signal amplification, electronic-skin interfaces, efficient transducers, POC testing, bioinformatics sharing along with rapid analysis to decide on therapy, and telemedicine are highly significant and certainly are improving the quality of life (Figure 1). A successful example of this is the biosensor-enabled COVID-19 pandemic management system [2,3,4], says Prof. B.D. Malhotra.

## Figures and Tables

**Figure 1:**
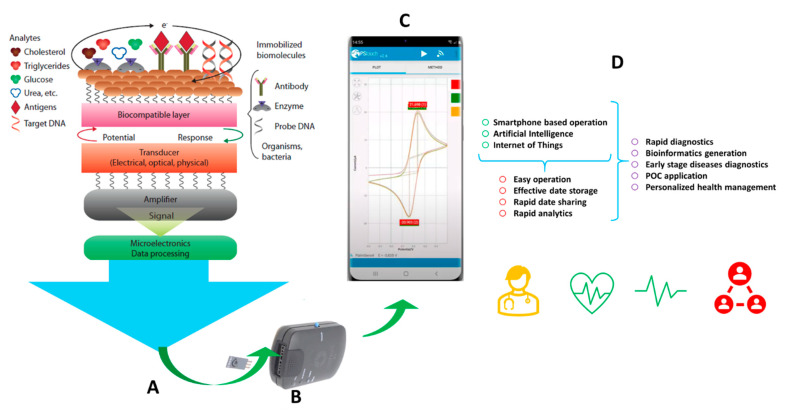
Illustration of a nano-enabled biosensing prototype for an efficient diagnostic. (**A**) Schematic presentation of a biosensing procedure involving selection and optimization of biomarkers, biorecognition molecules, nano-enabled biosensing substrates, and microelectronics; (**B**) Miniaturized electrode integrated with a miniaturized potentiostat; (**C**) Smartphone-enabled biosensing useful for POC applications; (**D**) Support of AI and IoMT for intelligent healthcare management.

## Data Availability

Not applicable.
